# Retraction: Early detection and classification of Alzheimer's disease through data fusion of MRI and DTI images using the YOLOv11 neural network

**DOI:** 10.3389/fnins.2026.1845919

**Published:** 2026-04-10

**Authors:** Frontiers Editorial Office

The journal retracts the March 11 2025 article cited above.

Following publication, concerns were raised regarding the scientific validity of the article. An investigation was conducted in accordance with Frontiers' policies.

The authors failed to provide a satisfactory explanation and as a result, the conclusions of the article have been deemed unreliable, and the article has been retracted.

This retraction was approved by the Chief Editors of Frontiers in Neuroscience and the Chief Executive Editor of Frontiers. The authors received a communication regarding the retraction and had a chance to respond. This communication is recorded by the publisher.

